# Ketamine as an Analgesic Adjunct for Opioid-Induced Hyperalgesia in a Patient With a Sickle Cell Pain Episode

**DOI:** 10.31486/toj.22.0011

**Published:** 2022

**Authors:** Michael R. Martinez, Emily H. Garmon, Garrett D. Starling, Monish A. Sheth

**Affiliations:** ^1^ Department of Anesthesiology, Baylor Scott & White Health, Texas A&M University College of Medicine, Temple, TX; ^2^ Department of Hospital Medicine, Baylor Scott & White Health, Texas A&M University College of Medicine, Temple, TX

**Keywords:** *Analgesics–opioid*, *anemia–sickle cell*, *hyperalgesia*, *ketamine*

## Abstract

**Background:** Ketamine is a noncompetitive N-methyl-D-aspartate receptor antagonist that has been proposed as a safe and effective nonopioid analgesic when given in lower doses than those historically used for general anesthesia. Case reports have demonstrated efficacy using low-dose ketamine for pain management and opioid weaning in patients with chronic noncancer pain, but reports of successful use in patients with sickle cell pain are limited.

**Case Report:** A 35-year-old African American male with sickle cell disease presented to the emergency department with severe generalized body aches and left flank pain. Several days later, his pain became localized to the bilateral lower extremities. Escalating opioid doses provided no improvement. Workup was negative for infection, deep venous thrombosis, ischemia, and infarct. On hospital day 29, the Acute Pain Management Service was consulted and initiated a low-dose ketamine infusion for analgesia and to facilitate opioid weaning. Five days later, the patient was discharged pain-free.

**Conclusion:** Ketamine is a potent nonopioid analgesic, and this report adds to the body of literature supporting the use of low-dose ketamine in patients with sickle cell disease to treat poorly controlled pain and opioid-induced hyperalgesia.

## INTRODUCTION

Sickle cell disease is a group of genetically inherited hemoglobinopathies in which hypoxemia causes an abnormal sickle morphology of red blood cells (Hb_s_). The shortened lifespan of Hb_s_ leads to anemia, while the accumulation of Hb_s_ molecules leads to clumping and occlusion of the capillary beds. Decreased end organ perfusion, ischemia, and infarction result in pathologic conditions associated with acute, subacute, and chronic pain.^1^

N-methyl-D-aspartate (NMDA) receptor activation is involved in the pathobiology of inflammation, nociceptive and neuropathic pain, opioid tolerance, opioid-induced hyperalgesia, and central sensitization.^1-3^ The same mechanisms have been implicated in sickle cell pain.^1^ Because of the diverse etiology of sickle cell pain, multimodal treatment strategies are recommended, with particular emphasis on patient-controlled opioid analgesia, which is associated with lower cumulative opioid requirement than intermittent administration.^4,5^ Other supportive measures include intravenous (IV) fluids, blood transfusions, and hydroxyurea.^6^

Ketamine, a noncompetitive NMDA receptor antagonist that has historically been used for sedation and general anesthesia at doses of 1 to 4.5 mg/kg,^7^ has been proposed as a safe and effective nonopioid analgesic when given in lower doses.^8,9^ Case reports have demonstrated efficacy using low-dose ketamine for pain management and opioid weaning in chronic noncancer pain.^10,11^ Although ketamine has been proposed for the treatment of sickle cell pain, case reports demonstrating profound analgesia and rapid opioid taper in sickle cell pain are limited.^3,4,12,13^

## CASE REPORT

A 35-year-old African American male with a medical history significant for asthma, previous tobacco abuse, and known sickle cell disease presented to the emergency department with severe generalized body aches, lower back pain, left flank and hip pain, and dyspnea, consistent with multiple prior sickle cell crises. His most recent admission for a similar episode was 6 months prior, at which time he was prescribed hydroxyurea 500 mg twice daily (180 capsules, 1 refill) and hydromorphone 4 mg orally every 4 hours as needed (42 tablets, no refills). He reportedly ran out of his hydroxyurea prescription 2 days prior to the current presentation because he did not establish care with a hematologist as had been recommended.

The patient complained of fatigue, arthralgias, and the myalgias mentioned above. His initial vital signs were within normal limits except for an oxygen saturation of 88% on room air that normalized with supplemental oxygen via nasal cannula. On physical examination, the patient was noted to be in distress, clutching his left hip, and was tender to palpation along the left flank and lower extremity. He was also noted to have scleral icterus. Abnormal admitting laboratory values included hemoglobin of 7.4 g/dL (reference range, 14.0-18.0 g/dL), reticulocyte count of 20.2% (reference range, 0.5%-2.2%), total bilirubin of 12.5 mg/dL (reference range, 0.2-1.2 mg/dL), and lactate dehydrogenase of 1,151 U/L (reference range, 84-246 U/L).

The patient's initial inpatient treatment regimen consisted of dextrose 5% with 0.45% normal saline maintenance IV fluids at 125 mL per hour, folic acid 1 mg per day, acetaminophen 325 mg every 4 hours, oral hydromorphone 4 mg every 6 hours scheduled, and IV hydromorphone 1 to 2 mg every 2 hours as needed for breakthrough pain. On hospital day 2, a hydromorphone patient-controlled analgesia infusion was initiated with a basal rate of 0.5 mg per hour and 0.4 mg every 10 minutes by demand dose. Despite aggressive treatment, the patient's anemia worsened, and he required 2 transfusions during the next week to maintain a hemoglobin concentration >6 mg/dL (nadir was 5.5 mg/dL on hospital day 3). By hospital day 3, the patient's flank pain was replaced by progressively worsening bilateral lower extremity pain, without associated skin changes, ulceration, erythema, or signs of obvious infection. Despite optimal nonopioid analgesics (celecoxib 200 mg twice daily and acetaminophen 325 mg every 4 hours), he required escalating doses of oral hydromorphone (from 4 mg per day on hospital day 2 to 16 mg per day by hospital day 3) and IV hydromorphone by patient-controlled analgesia (from an average of 35 mg per day on hospital day 3 to an average of 80 mg per day by hospital day 20). At the peak of the patient's inpatient opioid requirement, he used more than 1,200 mg morphine equivalent daily dose.^14^ Gabapentin 300 mg 3 times daily was added to the patient's nonopioid analgesic regimen with no improvement in his pain scores.

Extensive workup for his bilateral lower extremity pain included negative venous and arterial lower extremity Doppler ultrasound studies, negative bilateral knee and ankle joint synovial fluid analysis, and an equivocal magnetic resonance imaging scan suggestive of remote left tibial and fibular infarction vs chronic venous stasis.

By the third week of hospitalization, the patient's anemia and swelling had improved with IV furosemide 20 mg per day as needed, but he was unable to meet discharge criteria because of severe pain. The hospital Acute Pain Management Service was consulted on hospital day 29. On evaluation, the patient reported his pain level to be 10 of 10 on the visual analog scale in his bilateral ankles, radiating to his knees. Physical examination demonstrated bilateral ankle edema and tenderness to palpation in the bilateral lower extremities but otherwise no abnormalities (Figure). His pain was believed to be multifactorial, with the primary underlying cause being persistent swelling in his ankles that was complicated by opioid-related hyperalgesia from prolonged high-dose IV opioids during his hospital stay. His vaso-occlusive crisis was believed to be completely resolved and not contributing to his pain.

**Figure.**
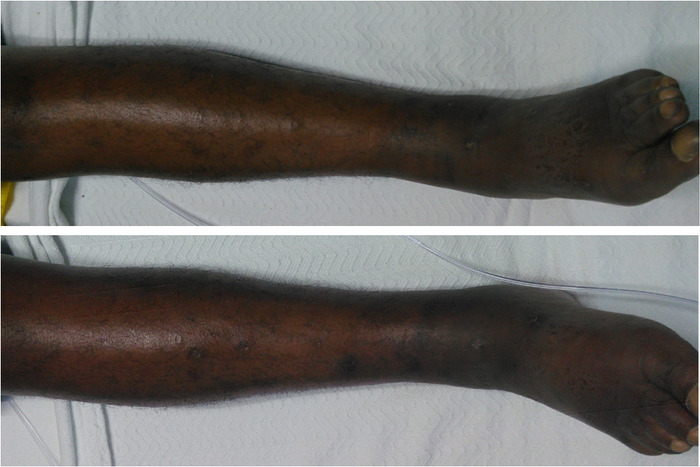
Chronic bilateral lower extremity cutaneous changes in the presence of sickle cell disease with edema noted on initial physical examination by Acute Pain Management Service.

A 5-day opioid weaning schedule was recommended to facilitate discharge, along with frequent Clinical Opiate Withdrawal Scale assessments to monitor for opioid withdrawal symptoms.^15^ Nonopioid analgesics were continued with slight dosage adjustments to optimize analgesia and minimize adverse effects, and a continuous ketamine infusion was initiated and titrated to a maximum dose of 0.4 mg/kg/h during the next 4 days (Table). IV furosemide 20 mg daily was also continued to facilitate diuresis and reduce edema. The patient tolerated the opioid-weaning schedule with improved pain and swelling. As planned, the patient was discharged on hospital day 34 with slight escalations of nonopioid analgesic doses and oral opioid dosage for breakthrough pain that was lower than the dose he had received after his prior hospitalization. His regimen included hydroxyurea 500 mg twice daily (180 capsules, 3 refills), folic acid 1 mg daily (90 tablets, 3 refills), acetaminophen 1,000 mg every 6 hours (over the counter), gabapentin 300 mg every 8 hours (90 capsules, no refills), and hydromorphone 2 mg every 6 hours as needed for breakthrough pain (60 tablets, no refills). His reported pain score at discharge was 0 of 10 on the visual analog scale. He was scheduled for 1-week follow-up to encourage improved chronic disease management in the outpatient setting.

**Table.** Patient's Medication Regimen After Ketamine Addition and Associated Down-Trending Pain Scores

	Day of Ketamine Administration				
Medication/Pain Score	1	2	3	4	5
Ketamine infusion, mg/kg/h	0.1	0.2	0.3	0.4	–
Hydromorphone infusion, mg/h	1	0.5	0.25	–	–
Oral hydromorphone, mg every 6 h	1	2	4	4	2
Gabapentin, mg every 8 h	300	300	300	300	300
Daily pain scores	8	6	4-5	0-3	0

Note: Not shown are scheduled intravenous furosemide 20 mg daily, acetaminophen 1,000 mg every 6 hours, and celecoxib 100 mg twice daily.

## DISCUSSION

Episodes of intractable pain are often multifactorial, with the interplay between somatic and psychological aspects manifesting in a complex clinical presentation.^16^ In this case, the pain episode was precipitated by a social issue (ie, the patient's failure to follow up, leading to his inability to continue chronic preventive therapy). Why the patient chose not to establish care, despite sufficient time and resources to do so, is unclear. His outcome illustrates the importance of multidisciplinary treatment teams for chronic progressive medical conditions such as sickle cell disease.^1,4^ Optimal outcomes occur when care is coordinated between primary care and specialists such as hematologists and others as indicated for symptom management.^6,17^ Palliative care is particularly helpful to establish long-term goals of care and optimal pain management. In addition, mental health professionals (both psychologists and psychiatrists) can be instrumental in managing depression, anxiety, and other psychological aspects of the disease. The psychosocial issue precipitating this patient's ineffective symptom management was addressed by having the patient scheduled for close follow-up in the clinic at discharge.

With regard to the somatic aspects of this patient's pain exacerbation, his initial flank and hip pain were consistent with his previous episodes of vaso-occlusive crisis. However, the etiology of his severe pain at admission was not the cause of his ongoing poorly controlled pain and progressively increasing opioid requirement during the hospital stay. His initial pain resolved shortly into his hospitalization and evolved into lower extremity pain that was unresponsive to increasing opioid analgesics. Detection of this shift in pain from the vaso-occlusive crisis to pain secondary to acute venous stasis in the setting of volume overload and localized tissue stretch was challenging because of the subtle changes he described. The patient's unusually high pain scores were inconsistent with scores typically reported from swelling alone and prompted the consideration of concomitant psychological factors, opioid tolerance requiring escalating doses for similar effect, or opioid-induced hyperalgesia resulting from unusually high opioid doses. The patient was otherwise completely reasonable in his complaints, demonstrating adequate coping strategies, and he had no other identifiable psychosocial issues complicating his recovery.

Tolerance vs opioid-induced hyperalgesia was challenging to discern. These distinct phenomena lead to the clinical experience of worsened pain, but tolerance results from loss of treatment effect (desensitization of opioid antinociceptive effect), whereas opioid-induced hyperalgesia results from sensitization of pronociceptive pathways.^2^ Although not completely understood, opioid-induced hyperalgesia is a complex process involving neuroplastic changes in the peripheral and central nervous systems, in part because of NMDA receptor activation.^2^ Ketamine, a noncompetitive NMDA receptor antagonist and potent analgesic, is believed to reverse pronociceptive pathway sensitization, making it an ideal therapy for paradoxical situations in which a patient becomes more sensitive to pain despite increasing doses of opioids.^10^ In addition, low-dose ketamine has been shown to be safe and effective at modulating central sensitization, hyperalgesia, and opioid tolerance, as well as facilitating rapid opioid weaning.^9,10^ Case reports and retrospective case series about patients with opioid-induced hyperalgesia and sickle cell vaso-occlusive crises have also demonstrated benefit.^3,12^ Lack of familiarity with ketamine, along with its reputation as an anesthetic, has limited broader use by some clinicians as a low-dose infusion; however, its safety profile at subanesthetic doses (<0.5 mg/kg/h) in the general ward setting is well documented in the literature.^7^ Admission to an intensive care unit for administration of this medication at subanesthetic doses is not routinely necessary but is a common practice because of the risk of psychomimetic and cardiovascular side effects reported with higher doses. Hospitals with well-defined protocols that allow for administration of subanesthetic doses in general ward settings are able to optimize cost and resources, especially in the context of the coronavirus disease 2019 pandemic. Ketamine may also confer advantages of preserved spontaneous respiration and bronchodilation, as opposed to opioid-induced respiratory depression. In addition, ketamine may promote hemodynamic stability in patients with normal catecholamine stores by releasing endogenous norepinephrine.^18-20^

## CONCLUSION

This case adds to the body of literature supporting the safety and efficacy of low-dose ketamine in patients with sickle cell disease for treatment of poorly controlled pain and opioid-induced hyperalgesia. Our patient not only achieved complete pain resolution with low-dose ketamine but also was able to wean from a high dose of morphine equivalents during a 5-day taper, facilitating discharge. Studies are needed to determine the optimal use of low-dose ketamine in patients with sickle cell disease.

## Notes

*This article meets the Accreditation Council for Graduate Medical Education and the American Board of Medical Specialties Maintenance of Certification competencies for Patient Care and Medical Knowledge.*
